# Endoscopic treatment of a patient with duodenal major papilla adenoma and ansa pancreatica

**DOI:** 10.1002/deo2.240

**Published:** 2023-05-08

**Authors:** Yan‐yan Wu, Yun‐lu Feng, Ai‐ming Yang

**Affiliations:** ^1^ Department of Gastroenterology Peking Union Medical College Hospital, Chinese Academy of Medical Sciences & Peking Union Medical College Beijing China

**Keywords:** ansa pancreatica, duodenal major papilla adenoma, endoscopic retrograde cholangiopancreatography, hybrid endoscopic mucosal resection, recurrent pancreatitis

## Abstract

A 35‐year‐old female who suffered from recurrent pancreatitis was admitted to our hospital. Her magnetic resonance cholangiopancreatography revealed ansa pancreatica. And during endoscopic retrograde cholangiopancreatography, a major duodenal papilla adenoma was identified. Hybrid endoscopic mucosal resection of this lesion was performed with pancreatic stent placement through the minor papilla to prevent recurrent pancreatitis. To our knowledge, this is the first report of a major papilla adenoma associated with ansa pancreatica. These minimally invasive endoscopic treatments solved a difficult clinical problem and avoided traumatic surgery.

## INTRODUCTION

Anatomic variations in the pancreatic duct can contribute to recurrent pancreatitis. Ansa pancreatica is an uncommon ductal anomaly with a reported prevalence of 0.5%–1.1%.^1^ Tumors arising in the region of the major duodenal papilla account for 5% of gastrointestinal neoplasms and are most commonly ampullary adenomas.^2^, ^3^ Here we reported a patient presented with recurrent acute pancreatitis. She was diagnosed with ansa pancreatica as well as duodenal major papilla adenoma. She underwent therapeutic endoscopy and recovered well.

## CASE REPORT

**FIGURE 1:**
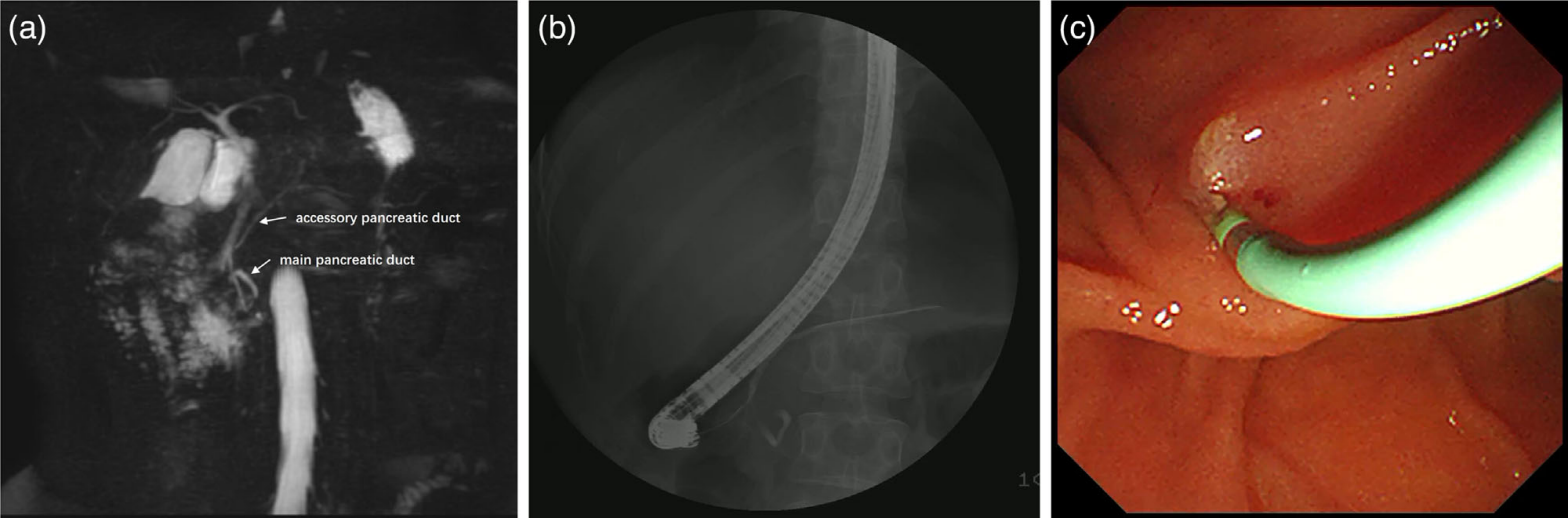
Magnetic resonance cholangiopancreatography and endoscopic retrograde cholangiopancreatography of the patient: (a, b) Magnetic resonance cholangiopancreatography and pancreaticography showed an accessory pancreatic duct draining into a minor papilla and looping of the proximal part of the main pancreatic duct. (c) A pancreatic stent was placed into the dorsal pancreatic duct through the minor papilla.

A 35‐year‐old woman reported suffering from recurrent epigastric pain for 5 years and was diagnosed with recurrent acute pancreatitis previously. She had a negative past medical history and a family medical history. She didn't smoke and took occasional alcohol. Laboratory examination revealed significantly elevated serum amylase and lipase levels (amylase 852U/L and lipase 1311U/L). Bilirubin, serum calcium, triglyceride, serum immunoglobulin G, and carbohydrate antigen 19‐9 were normal. Abdominal computed tomography suggested an edematous pancreas with no significant dilation of pancreatic ducts. A followed magnetic resonance cholangiopancreatography revealed an accessory pancreatic duct (of Santorini) draining into a minor papilla and looping of the proximal part of the main pancreatic duct (of Wirsung; Figure 1a).

**FIGURE 2:**
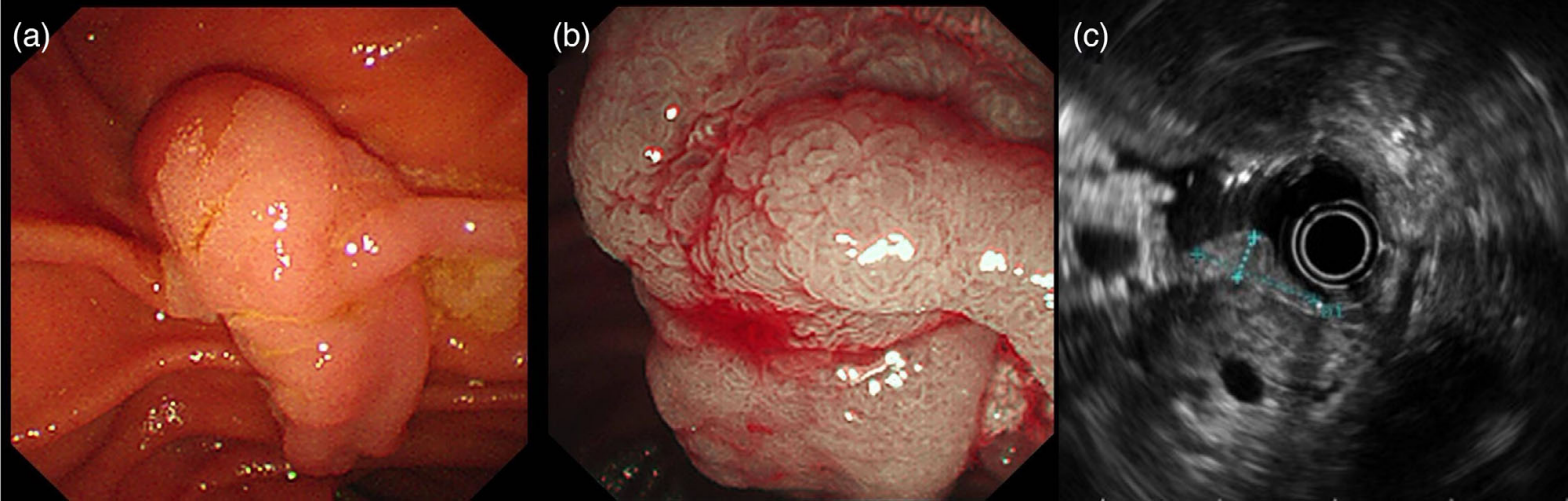
A major duodenal papilla lesion was identified: (a, b) A major duodenal papilla lesion was identified during endoscopic retrograde cholangiopancreatography. (c) Endoscopic ultrasonography showed intact submucosa to a depth of 7 mm.

**FIGURE 3:**
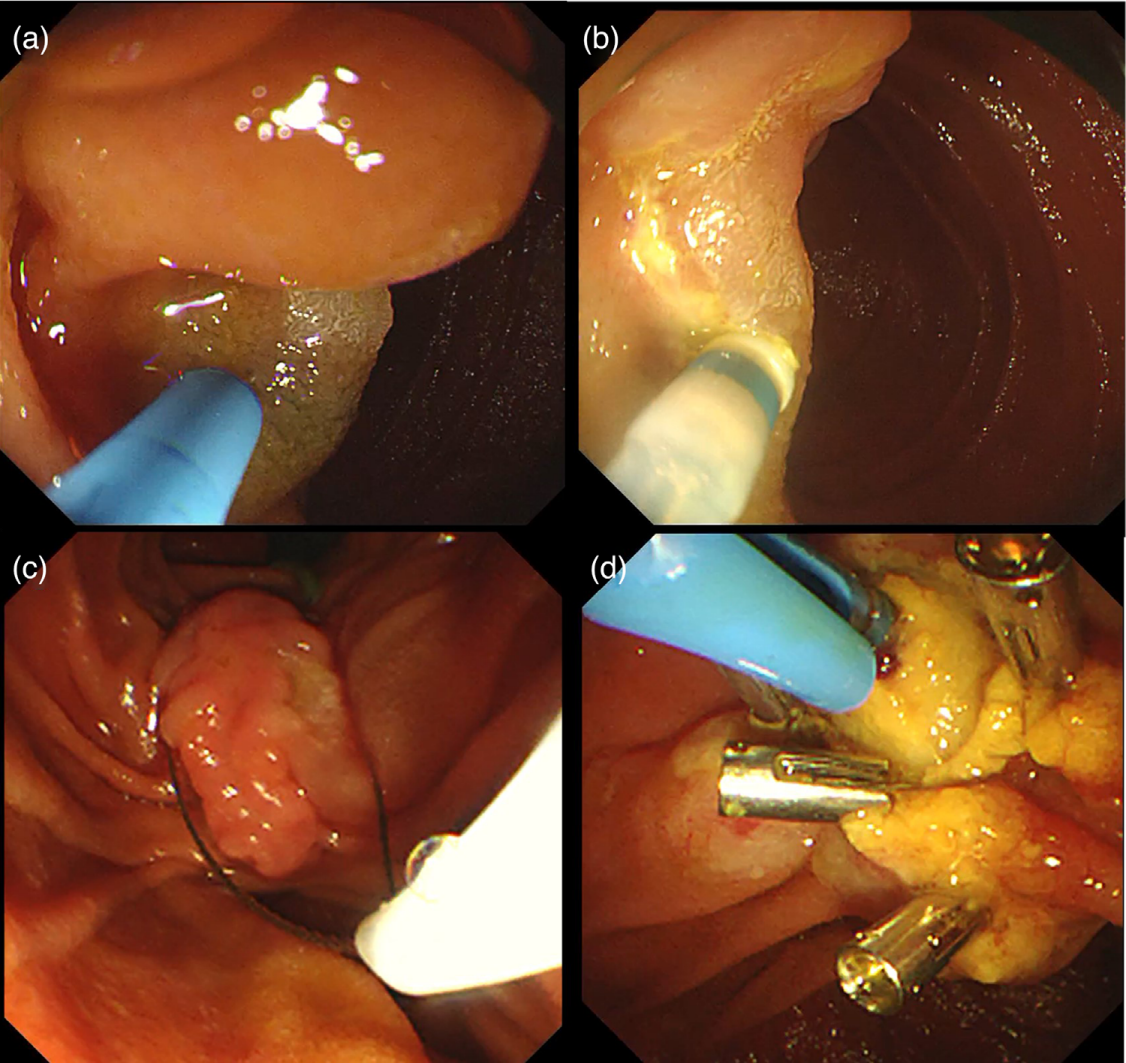
Hybrid endoscopic mucosal dissection of the duodenal major papilla adenoma: (a) A submucosal cushion was created by injection into the anterior, posterior, and oral sides of the lesion. (b) A halfway circumferential incision was made with a dual knife. (c) An en bloc resection with a snare. (d) A 5 cm 8 Fr biliary stent was inserted and hemostasis was performed by using endoclips.

Then the patient underwent endoscopic retrograde cholangiopancreatography (ERCP). Pancreaticography reconfirmed the anatomic variation of the pancreatic duct and the patient was diagnosed as ansa pancreatica (Figure 1b). An 11 cm 5 Fr pancreatic stent was placed into the dorsal pancreatic duct through the minor papilla (Figure 1c and Video S1). However, a major duodenal papilla lesion was identified during ERCP, and histopathological examination of a forceps biopsy suggested a probable tubular adenoma (Figure 2a,b). Endoscopic ultrasonography showed intact submucosa with a depth of 7 mm and no intraductal extension (Figure 2c). A month later, hybrid endoscopic mucosal dissection (ESD) was performed: a submucosal cushion was created by injection of a mixture of saline solution, methylene blue, and adrenaline into the anterior, posterior, and oral sides of the lesion, a halfway circumferential incision was made with a dual knife, and the lesion had an en bloc resection with a snare (Figure 3a–c). A 5 cm 8 Fr biliary stent was then inserted to prevent postoperative complications. Hemostasis was performed carefully by using endoclips (Figure 3d and Video S2). Histopathological examination confirmed the presence of a completely resected 12 × 7 mm tubulovillous adenoma (the endoscopes and devices models were provided in Table S1). The biliary stent was removed four weeks later, and the patient had no recurrent pancreatitis after the ERCP procedure. A year later, the patient underwent another endoscope for reexamination and found that the pancreatic duct migrated.

## DISCUSSION

Ansa pancreatica was first described by Dawson and Langman in the anatomic study of cadaveric subjects in 1961. It is suggested that inadequate drainage through the minor papilla may lead to recurrent acute pancreatitis in the setting of ansa pancreatica.^4^, ^5^ But the association was not solid since ansa pancreatica can also be identified in the asymptomatic general population.^1^ This patient had a major duodenal papilla adenoma at the same time. Acute pancreatitis owing to the periampullary tumor has been reported in previous literature and the obstruction of the major papilla was supposed to be the reason.^6^ But in this case, no dilation of the pancreatic duct due to obstruction was identified, and she had not experienced an episode of acute pancreatitis ever since ERCP. Thus, we considered that ansa pancreatica mainly accounted for her recurrent pancreatitis. In this case, we failed to cannulate through the major papilla during ERCP and only placed the pancreatic stent through the minor papilla. And the patient hadn't suffered from acute pancreatitis since then. So we supposed that the drainage through the main pancreatic duct was adequate. And the accessory pancreatic stent was enough for the treatment of her recurrent acute pancreatitis.

There is no established consensus for the endoscopic management of duodenal papillary lesions until now. Conventional endoscopic techniques such as snare polypectomy or EMR may lead to incomplete resection, tumor recurrence, and even surgical incision involved.^2^ Due to the complex anatomical structure of the duodenal papilla, ESD can be difficult technically. Wang et al. have reported their successful treatment of two patients with recurrent duodenal papillary adenomas by hybrid ESD combined with ERCP.^7^ In our case, we chose to perform hybrid‐ESD because we found that the major duodenal papilla lesion had a flat edge. Hybrid ESD was performed carefully by an experienced endoscopist, and the lesion was resected completely with a snare. Hemostasis was done with caution by endoclips. No severe adverse events such as bleeding, perforation, or injection pancreatitis occurred.

To our knowledge, this is the first report of a major papilla adenoma associated with ansa pancreatica. We performed hybrid ESD of the lesion with pancreatic stent placement through the minor papilla to prevent recurrent pancreatitis. These minimally invasive endoscopic treatments solved a difficult clinical problem and avoided traumatic surgery.

## CONFLICT OF INTEREST STATEMENT

None.

## Supporting information

Table S1: Endoscopes and devices models

Video S1: Endoscopic retrograde cholangiopancreatography and placement of a pancreatic stent.

Video S2: Hybrid endoscopic mucosal resection of the duodenal major papilla adenoma.
