# Supplementary material for: Endoscopic treatment of a patient with duodenal major papilla adenoma and ansa pancreatica
Source: DEN Open. 2023 May 8;4(1):e240. doi: 10.1002/deo2.240 (PMC10167413; doi:10.1002/deo2.240)
Supplement: Supplementary file 1 — Table S1: Endoscopes and devices models [file DEO2-4-e240-s001.docx]

**Supplementary table**

**Table 1 Endoscopes and devices models**

| **Endoscopes and devices** | **Models** |
| --- | --- |
| Endoscopic system | EVIS LUCERA ELITE CV-290 |
| Duodenoscope | JF-260V |
